# Aligning data with decisions to address the biodiversity crisis

**DOI:** 10.1371/journal.pbio.3002683

**Published:** 2024-06-11

**Authors:** Leah R. Gerber, Gwenllian D. Iacona

**Affiliations:** Center for Biodiversity Outcomes and School of Life Sciences, Arizona State University, Tempe, Arizona, United States of America

## Abstract

The planetary outlook for biodiversity is dire. This editorial introduces a new collection of articles that discuss the data we have and the data we need for more effective conservation policies, as well as the role that social justice and a multiple-stakeholder view have in ensuring a more sustainable future for our planet.

## Teaser

The planetary outlook for biodiversity is dire. A new collection of articles discusses the disconnect between the data we have and the data we need for more effective action on conservation, as well as how social justice and end-user viewpoints must be centered to ensure a more sustainable future for our planet.

Biodiversity is a cornerstone of ecosystem resilience and human well-being, yet faces unprecedented anthropogenic threats. The planetary outlook for biodiversity resilience and recovery remains dire, despite the efforts of conservation biologists to address the biodiversity crisis since the 1980s [1]. The lessons of preceding decades suggest that the interventions needed to achieve the desired biodiversity outcomes require system-wide and interdisciplinary efforts [2], and there have been urgent calls to rapidly improve species resilience and recovery across the globe (Secretariat of the Convention on Biological Diversity. Global Biodiversity Outlook 5). However, the path towards effective biodiversity conservation requires more than just good intentions; it demands action that stems from data-driven strategies and evidence-based decision-making [3].

Nevertheless, biological and ecological data alone do not result in biodiversity conservation. This is because conservation is a human decision-making problem; most human choices impact biodiversity outcomes, and conservation success depends on what we identify as important and the decisions we make to achieve stated goals [4,5]. People consistently rank environmental protection as a priority [6] but, to date, human decision-making has fallen short on considering the biodiversity consequences of different choices. These decisions might be about land use change, economic development, business practices, or policy development. If they result in consequences for environmental quality or species persistence, they implicitly influence biodiversity.

As we confront escalating challenges to protect biodiversity, it is crucial to ensure that rigorous and relevant data guide our decisions and actions [4]. Considerable data that would be relevant for biodiversity decisions has been compiled, but is not necessarily used for decision-making in ways that promote better outcomes [7], as discussed by Hawkins in this issue [8]. Decision-makers are often not equipped to determine the biodiversity outcomes of their choices or what data are most relevant to the decision at hand (e.g., [9]).

We contend that approaches to data generation and use for biodiversity conservation need to be turned on their head. By focusing on desired outcomes, we can clearly identify what information is needed for relevant decision-making. With outcomes articulated, decision-makers can then engage in a use-informed approach to designing conservation science and research initiatives that can support these goals. Supporting biodiversity-centered decision-making may require new and different data types and sources, and may even require completely different ways of doing science, but the field is starting to move in that direction.

In this issue of *PLOS Biology*, we have curated a collection of Perspectives and Essays under the theme of “Bringing data to decision making for conservation and biodiversity” to illuminate some data-to-decisions considerations important for biodiversity conservation. In many cases, while data-driven decision support approaches can improve conservation efficiency, the application of these approaches has been stymied by steep learning curves and difficulty in access to and analysis of data [10]. Conservation organizations tend to have staffs who are already stretched thin, and they cannot risk time and resources in finding and trialing new procedures [11]. This capacity challenge perpetuates what we call the data-for-decisions gap. To address the extinction crisis, we must not only develop cutting-edge tools, data, and analyses, but also collaborate with those who can apply them on the ground to improve conservation effectiveness, at a global scale.

The collection features a thought-provoking piece, provocatively titled “How will better data (and better use of data) enable us to save the planet?” [8] where Hawkins challenges us to align environmental and biodiversity data analytics and emerging technologies with the questions and decision-making necessary to address pressing environmental issues incurred and faced by private industry.

Adams contributes a critical examination of the data currently available to support decisions regarding the financial aspects of enacting conservation in “Costs in conservation: common costly mistakes and how to avoid them” [12]. Adams analyzes pitfalls and misconceptions surrounding the data appropriate for describing conservation expenditures and offers advice on ways forward. This article serves as a practical guide for conservation scientists and practitioners who are striving to support decisions regarding investment into conservation action and who want to optimize resource allocation for maximum conservation impact.

In “Now is the time for conservationists to stand up for social justice,” Milner-Gulland urges conservation scientists to confront the intersecting challenges of environmental degradation and social inequity [13]. By advocating for explicit consideration of justice and inclusivity in how data is collected and applied to biodiversity conservation issues, this Perspective emphasizes the interconnectedness of environmental and social issues. This article challenges us to ensure that data and decision-making work to dismantle systemic barriers to societal and conservation progress.

Finally, Ripple and colleagues [14] advocate for collaborative and interdisciplinary approaches to achieving desired conservation outcomes in “Enabling usable science takes a community: using our roles as funders to catalyze change.” Recognizing the inherent complexity of environmental problems, the authors emphasize the importance of fostering a vibrant community of researchers, practitioners, and policymakers committed to co-creating actionable solutions informed by robust scientific evidence.

The journey towards data-driven conservation is not without its challenges. Ethical considerations, data limitations, and institutional barriers loom on the horizon. This collection seeks to foster a frank and open dialogue about the opportunities and obstacles facing the field. By confronting these issues directly, we provide a shared vision of a world where knowledge informs decisions so that we can begin to chart a course towards a more sustainable and equitable future for all life on Earth.
